# Resveratrol Inhibits LPS-Induced MAPKs Activation via Activation of the Phosphatidylinositol 3-Kinase Pathway in Murine RAW 264.7 Macrophage Cells

**DOI:** 10.1371/journal.pone.0044107

**Published:** 2012-08-31

**Authors:** Yi Zong, Lin Sun, Bin Liu, Yi-Shu Deng, Dong Zhan, Yuan-Li Chen, Ying He, Jing Liu, Zong-Ji Zhang, Jun Sun, Di Lu

**Affiliations:** 1 Department of Anatomy, Kunming Medical University, Kunming, Yunnan, People’s Republic of China; 2 Department of Cardiology, The Second Affiliated Hospital, Kunming Medical University, Kunming, Yunnan, People’s Republic of China; 3 Department of Spine Surgery, The Third Affiliated Hospital, Sun Yat-sen University, Guangzhou, People’s Republic of China; 4 Department of Respiratory Medicine, The Third People’s of Yunnan Province, Kunming, Yunnan, People’s Republic of China; 5 Department of Pathology, Kunming Medical University, Kunming, Yunnan, People’s Republic of China; Fundação Oswaldo Cruz, Brazil

## Abstract

**Background:**

Resveratrol is a natural polyphenolic compound that has cardioprotective, anticancer and anti-inflammatory properties. We investigated the capacity of resveratrol to protect RAW 264.7 cells from inflammatory insults and explored mechanisms underlying inhibitory effects of resveratrol on RAW 264.7 cells.

**Methodology/Principal Findings:**

Murine RAW 264.7 cells were treated with resveratrol (1, 5, and 10 µM) and/or LPS (5 µg/ml). Nitric oxide (NO) and prostaglandin E2 (PGE2) were measured by Griess reagent and ELISA. The mRNA and protein levels of proinflammatory proteins and cytokines were analysed by ELISA, RT-PCR and double immunofluorescence labeling, respectively. Phosphorylation levels of Akt, cyclic AMP-responsive element-binding protein (CREB), mitogen-activated protein kinases (MAPKs) cascades, AMP-activated protein kinase (AMPK) and expression of SIRT1(Silent information regulator T1) were measured by western blot. Wortmannin (1 µM), a specific phosphatidylinositol 3-kinase (PI3-K) inhibitor, was used to determine if PI3-K/Akt signaling pathway might be involved in resveratrol’s action on RAW 264.7 cells. Resveratrol significantly attenuated the LPS-induced expression of nitric oxide (NO), prostaglandin E2 (PGE2), inducible nitric oxide synthase (iNOS), cyclooxygenase-2 (COX-2), tumor necrosis factor-α (TNF-α) and interleukin-1β (IL-1β) in RAW 264.7 cells. Resveratrol increased Akt phosphorylation in a time-dependent manner. Wortmannin, a specific phosphatidylinositol 3-kinase (PI3-K) inhibitor, blocked the effects of resveratrol on LPS-induced RAW 264.7 cells activation. In addition, PI3-K inhibition partially abolished the inhibitory effect of resveratrol on the phosphorylation of cyclic AMP-responsive element-binding protein (CREB) and mitogen-activated protein kinases (MAPKs) cascades. Meanwhile, PI3-K is essential for resveratrol-mediated phosphorylation of AMPK and expression of SIRT1.

**Conclusion and Implications:**

This investigation demonstrates that PI3-K/Akt activation is an important signaling in resveratrol-mediated activation of AMPK phosphorylation and SIRT1 expression, and inhibition of phosphorylation of CREB and MAPKs activation, proinflammatory mediators and cytokines production in response to LPS in RAW 264.7 cells.

## Introduction

Lipopolysaccharide (LPS), a main component of outer membrane of Gram-negative bacteria, has been referred to study experimentally induced infection, inflammation, or tissue damage, as well as the biochemistry of inflammatory responses. The studies have suggested that exposure of mammalian cells to LPS can lead to release of proinflammatory cytokines and in turn activate a second level of inflammatory cascades including cytokines, lipid mediators and adhesion molecules such as nitric oxide (NO), prostaglandin E2 (PGE2), tumor necrosis factor-α (TNF-α), interleukin-1β (IL-1β), reactive oxygen species (ROS), inducible nitric oxide synthase (iNOS), and cyclooxygenase-2 (COX-2) [1]. Recently, increasing evidence has revealed that LPS can induce the inflammatory response by activating numerous inflammatory cells and result in diabetes, chronic obstructive pulmonary disease, neurodegenerative diseases and osteoporosis [2], [3], [4], [5], [6].

Macrophages are important inflammatory cells implicated in the initiation of inflammatory responses, and play critical roles in the pathogenesis of numerous inflammatory disease processes by secreting various proinflammatory mediators and proinflammatory cytokines [7]. Therefore, modulation of macrophage-mediated inflammatory responses is important for creating a new therapeutic approach against these inflammatory diseases. Moreover, LPS can activate macrophages via multiple signaling pathways and, thus, enhance the production of proinflammatory mediators and proinflammatory cytokines. The mitogen-activated protein kinase (MAPK) signaling pathway in macrophages is one of the most extensively investigated intracellular signaling cascades involved in LPS-induced proinflammatory responses [8], [9], [10], [11], which are classified into at least three components: extracellular signal-regulated kinases 1/2 (ERK 1/2), c-Jun N-terminal kinase (JNK), and p38 MAPK and which have been implicated in the release of immune-related cytotoxic factors and proinflammatory cytokines [12], [13], [14]. AMP-activated protein kinase (AMPK) is a highly conserved heterotrimeric serine/threonine kinase that is a crucial regulator of energy metabolic homeostasis at the cellular and whole organism levels [15]. Recently, it has been found that AMPK/SIRT1 (Silent information regulator T1) pathway plays an important role in inflammation and can serve as a potential target to treat inflammation-related disorders [16].

**Figure 1:**
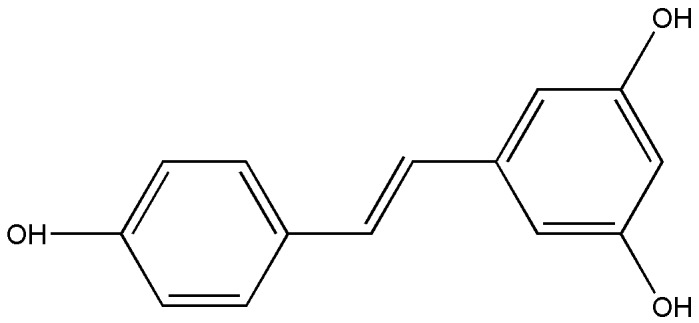
Chemical structure of resveratrol.

Resveratrol (3, 4, 5-trihydroxy-trans-stilbene) is a natural non-flavonoid polyphenolic found in grapes, red wine, mulberries, knotweed, peanuts and other plants (Figure 1) [17]. Although these plants and their extracts have been used for various therapeutic purposes by ancient cultures, resveratrol itself was first described in 1940 as a phenolic component of the medicinal herb hellebore [18]. There are numerous reports in the literature show that resveratrol dampens inflammation in arthritis and immune responsiveness in autoimmune diseases [19], [20], suppresses angiogenesis and metastasis in several cancers [21], and inhibits ROS products and platelet aggregation in cardiovascular diseases [22], [23]. Resveratrol can attenuate the activation of immune cells and the subsequent synthesis and release of proinflammatory mediators through the inhibition of the MAPK signaling pathway [24], [25], [26], [27]. However, the precise molecular mechanisms explaining how resveratrol suppresses the inflammatory response in macrophages are unknown.

Phosphatidylinositol 3-kinase (PI3-K), a dual protein and lipid kinase, has been shown to mediate signaling induced by numerous growth factors and tumour antigens. PI3-K is a heterodimeric complex consisting of a 85-kDa regulatory subunit, p85, and a 110-kDa catalytic subunit, p110 [28]. The activated receptor binds PI3-K, which then phosphorylates phosphatidylinositol, catalyzing the formation of phosphatidylinositol 3,4,5-triphosphate as the second messenger, using phosphatidylinositol 4,5-bisphosphate as the substrate. This activates downstream signaling molecules such as Akt. Activated Akt then dissociates from the membrane to act on its targets in the cytosol and the nucleus [29], and plays a crucial role in a wide variety of biological responses including cell survival, mitogenesis, and cell migration [30]. Recently, it has been showed that the PI3K/Akt signaling pathway plays an important role in negatively regulating LPS-induced acute inflammatory responses *in vitro* and *in vivo* [31], [32]. However, a recent study indicated that the anti-inflammatory effect induced by psoralidin *in vitro* on macrophages was associated with the inhibition of the PI3K/Akt pathway that blocks proinflammatory mediators and proinflammatory cytokines production [33].

This study examined the effects of resveratrol on LPS-stimulated inflammatory responses in macrophages and the potential role of PI3-K signaling in this process. To this end, a fuller understanding of the molecular mechanism of macrophage activation is clearly desirable in delineating the therapeutic target molecules to reduce the inflammatory responses in various inflammatory diseases.

## Results

### Resveratrol and/or Wortmannin do not Affect the Viability of RAW 264.7 Cells

**Figure 2:**
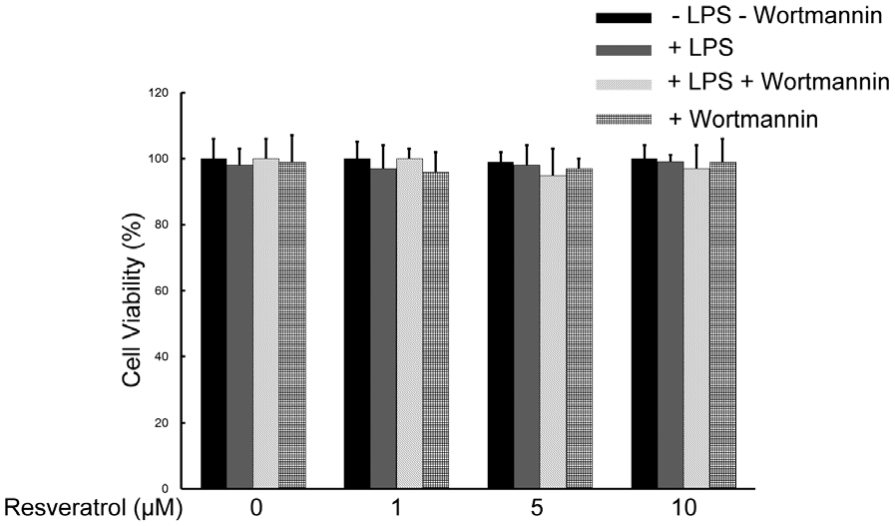
Effects of resveratrol on the cell viability of RAW 264.7 macrophage cells. RAW 264.7 cells were treated with 1, 5, and 10 µM of resveratrol without LPS treatments or with 5 µg/ml LPS or with 1 µM wortmannin treatments for 24 h. RAW 264.7 cells viability was measured and expressed as mean ± SEM for three independent experiments.

The cytotoxicity of resveratrol and the PI3-K inhibitor wortmannin were evaluated in the presence or absence of LPS by MTT assay. Resveratrol and/or wortmannin did not decrease the viability of the RAW 264.7 macrophage cells when they were incubated with or without LPS (5 µg/ml) in the presence or absence of resveratrol (1, 5, and 10 µM) and/or wortmannin (1 µM); hence, resveratrol and/or wortmannin exerted no significant cytotoxicity on RAW 264.7 macrophage cells (Figure 2).

### Resveratrol Activates the PI3-K/Akt Signaling Pathway in RAW 264.7 Cells

**Figure 3:**
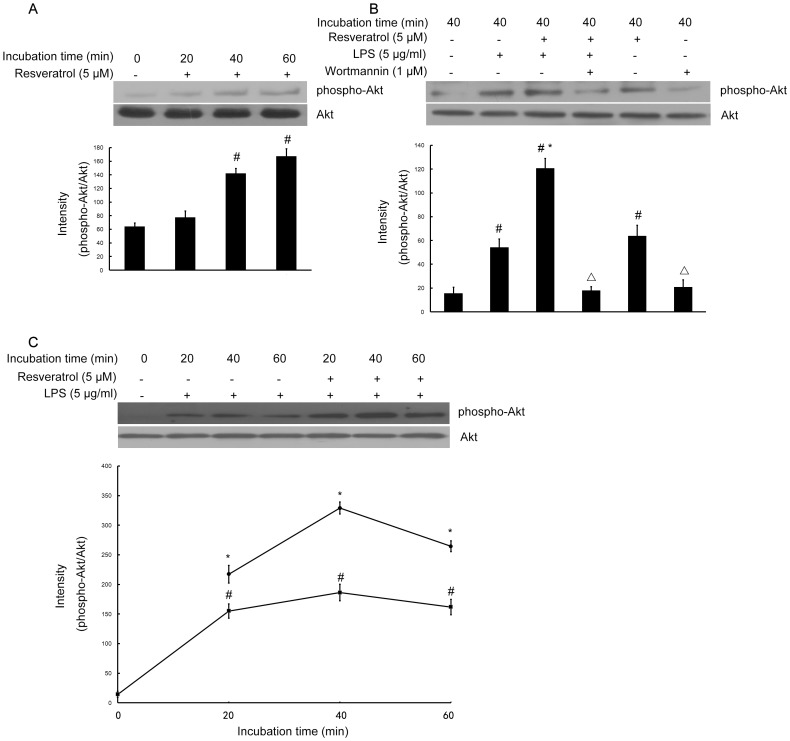
Resveratrol activates the PI3-K/Akt signaling pathway in RAW 264.7 macrophage cells. Panel A shows that RAW 264.7 cells were treated with resveratrol (5 µM) for the indicated times, Panel B shows that RAW 264.7 cells were pre-treated with resveratrol (5 µM) for 1 h in the absence or presence of wortmannin (1 µM), then exposed to LPS (5 µg/ml) for 40 min and Panel C shows that the cells were incubated with LPS (5 µg/ml) without or with 5 µM resveratrol for the indicated times. Various treated RAW 264.7 cell lysates (50 µg protein) were prepared and subjected to Western blot analysis by using antibodies specific for total Akt and phosphorylated forms of Akt (shown as phospho-Akt) as described in the methods. The relative protein levels were quantified by scanning densitometry and normalized to total Akt. The values shown are mean ± SEM of data from three independent experiments. ^#^Significant compared with control alone, *p*<0.05. ^*^Significant compared with LPS alone, *p*<0.05. ^Δ^Significant compared with resveratrol + LPS, *p*<0.05.

To identify the signaling pathways that are activated by resveratrol in cultured mouse macrophage cell line RAW 264.7 cells. We investigated whether resveratrol could activate the PI3-K/Akt pathway and affect LPS-induced Akt phosphorylation. Incubation of RAW 264.7 cells with resveratrol 5 µM significantly induced phosphorylation of serine residue 473 of Akt in time-dependent manner (Figure 3A). Furthermore, pretreatment of RAW 264.7 cells with resveratrol (5 µM) 1 h before LPS challenge (5 µg/ml) for 40 min enhanced Akt phosphorylation compared with LPS treatment alone. Interestingly, phosphorylation of Akt was inhibited by the PI3-K inhibitor wortmannin (1 µM), suggesting that reveratrol-induced activation of Akt is PI3-K dependent (Figure 3B).

We further examined whether resveratrol affects Akt phosphorylation induced by LPS. Incubation of RAW 264.7 cells with LPS (5 µg/ml) increased Akt phosphorylation in a time-dependent manner (Figure 3C). When the cells were treated with resveratrol (5 µM) for 1 h before LPS stimulation, Akt phosphorylation was elevated initially and further increased steadily during the 1 h incubation with LPS. The levels of phospho-Akt were always significantly higher in the cells pretreated with resveratrol compared with the cells treated with LPS only (Figure 3C).

### Resveratrol Inhibited Secretions of NO and PGE2 Induced by LPS via the PI3-K Pathway in RAW 264.7 Cells

**Figure 4:**
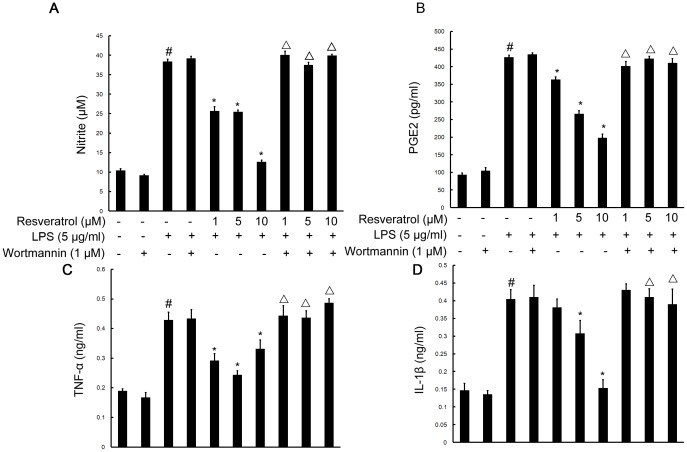
PI3-K is required for resveratrol-inhibited the production of NO, PGE2, TNF-α and IL-1β induced by LPS in RAW 264.7 macrophage cells. Approximately 1×10^6^ cells/ml were seeded in six-well plates and incubated until 80% confluency. Cells were pre-treated with resveratrol (1, 5, and 10 µM) for 1 h in the absence or presence of wortmannin (1 µM), then exposed to LPS (5 µg/ml) for 12 h. The concentrations of NO (A), PGE2 (B), TNF-α (C) and IL-1β (D) were measured as described in the methods. The values shown are mean ± SEM of data from three independent experiments. ^#^Significant compared with control alone, *p*<0.05. ^*^Significant compared with LPS alone, *p*<0.05. ^Δ^Significant compared with resveratrol + LPS, *p*<0.05.

To elucidate the contribution of PI3K/Akt pathway to the regulation of NO and PGE2 secretions, RAW 264.7 cells were pretreated with wortmannin (1 µM) for 1 h and then treated with LPS (5 µg/ml) and resveratrol (1, 5, and 10 µM) for 12 h. As shown in Figures 4A and B, treatment of RAW 264.7 cells with LPS caused a significantly increase in NO and PGE2 secretions in comparison with untreated controls by Griess reagent and enzyme-linked immunosorbent assay (ELISA). Resveratrol inhibited the LPS-induced production of NO and PGE2 over the concentration range used here. However, the inhibition of PI3-K with wortmannin indeed attenuated the suppression effects of resveratrol on NO and PGE2 secretions in RAW 264.7 cells (Figures 4A and B).

These data suggest that resveratrol suppresses NO and PGE2 secretions, at least in part, by PI3-K/activation in the LPS-treated RAW 264.7 cells.

### Resveratrol Inhibits LPS-stimulated Expression of iNOS and COX-2 Proteins and mRNA via Activation of the PI3-K Signaling Pathway in RAW 264.7 Cells

**Figure 5:**
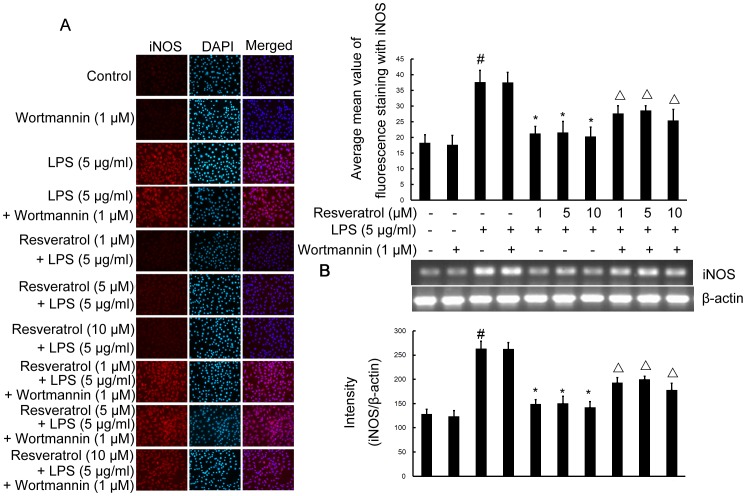
PI3-K is required for resveratrol-inhibited expression of iNOS protein and mRNA induced by LPS in RAW 264.7 macrophage cells. Panel A shows the immunofluorenscence images for protein expression of iNOS and Panel B shows the corresponding mRNA data. The relative mRNA level was quantified by scanning densitometry and normalized to β-actin mRNA. Note the up-regulated protein and mRNA expression of iNOS by LPS is suppressed by different concentrations of resveratrol; however, in cells pretreated with PI3-K inhibitor wortmannin, the suppressive effect of resveratrol is abrogated. The values shown are mean ± SEM of data from three independent experiments. ^#^Significant compared with control alone, *p*<0.05. ^*^Significant compared with LPS alone, *p*<0.05. ^Δ^Significant compared with resveratrol + LPS, *p*<0.05.

**Figure 6:**
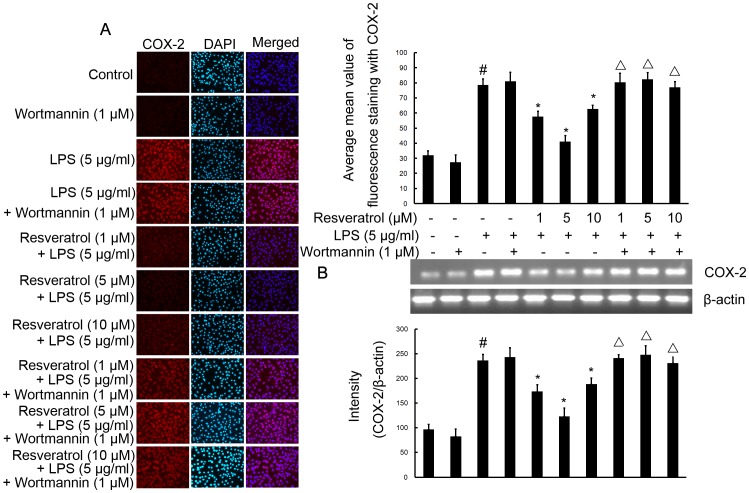
PI3-K is required for resveratrol-inhibited expression of COX-2 protein and mRNA induced by LPS in RAW 264.7 macrophage cells. Panel A shows the immunofluorenscence images for protein expression of COX-2 and Panel B shows the corresponding mRNA data. The relative mRNA level was quantified by scanning densitometry and normalized to β-actin mRNA. Note the up-regulated protein and mRNA expression of COX-2 by LPS is suppressed by different concentrations of resveratrol; however, in cells pretreated with PI3-K inhibitor wortmannin, the suppressive effect of resveratrol is abrogated. The values shown are mean ± SEM of data from three independent experiments. ^#^Significant compared with control alone, *p*<0.05. ^*^Significant compared with LPS alone, *p*<0.05. ^Δ^Significant compared with resveratrol + LPS, *p*<0.05.

To determine the relevance of the PI3-K signaling pathway on the ability of resveratrol to decrease iNOS and COX-2 expression in response to LPS challenge, RAW 264.7 cells were treated with wortmannin in the presence or absence of resveratrol. Pre-treatment cells with resveratrol (1, 5, and 10 µM) significantly inhibited iNOS and COX-2 protein and mRNA levels (Figures 5 and 6), compared with LPS (5 µg/ml)-treated cells for 6 h. Treatment of cells with wortmannin (1 µM) did not affect LPS-stimulated iNOS and COX-2 protein expression and mRNA levels. Wortmannin also did not affect LPS-stimulated iNOS and COX-2 protein expression and mRNA levels. However, wortmannin abolished the inhibitory effect of resveratrol on the protein and mRNA levels of iNOS and COX-2 in RAW 264.7 cells (Figures 5 and 6).

Thus, activation of PI3-K is essential for resveratrol-mediated inhibition of the protein and mRNA levels of iNOS and COX-2 in RAW 264.7 cells.

### PI3-K is Involved in Resveratrol-attenuated Expression of the Proinflammatory Cytokines TNF-α and IL-1β in LPS Stimulated RAW 264.7 Cells

**Figure 7:**
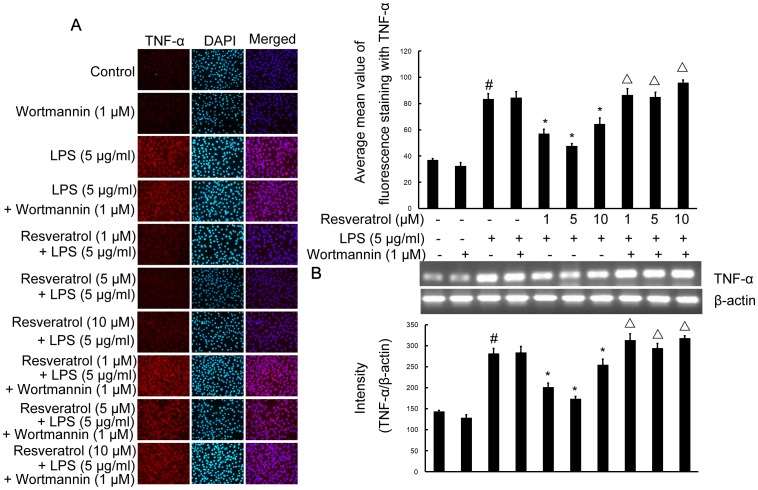
PI3-K is involved in resveratrol-attenuated the production of the proinflammatory cytokine TNF-α at the transcriptional and translational levels in RAW 264.7 macrophage cells. Panel A shows the immunofluorenscence images for protein expression of TNF-α and Panel B shows the corresponding mRNA data. The relative mRNA level was quantified by scanning densitometry and normalized to β-actin mRNA. Note the up-regulated protein and mRNA expression of TNF-α by LPS is suppressed by different concentrations of resveratrol; however, in cells pretreated with PI3-K inhibitor wortmannin, the suppressive effect of resveratrol is abrogated. The values shown are mean ± SEM of data from three independent experiments. ^#^Significant compared with control alone, *p*<0.05. ^*^Significant compared with LPS alone, *p*<0.05. ^Δ^Significant compared with resveratrol + LPS, *p*<0.05.

**Figure 8:**
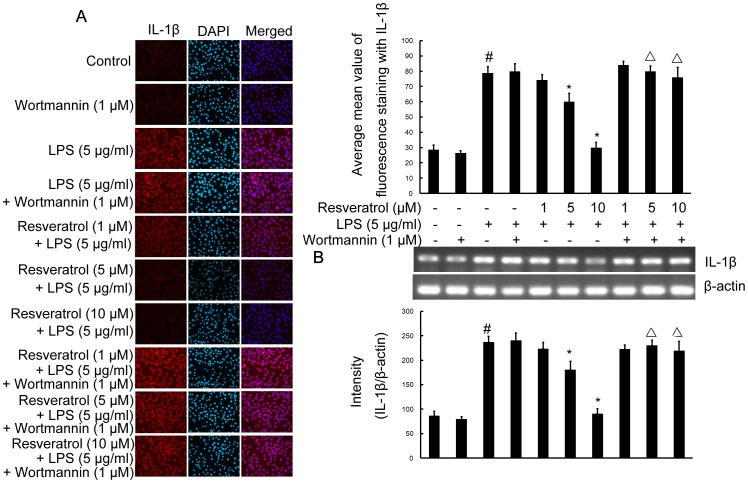
PI3-K is involved in resveratrol-attenuated the production of the proinflammatory cytokine IL-1β at the transcriptional and translational levels in RAW 264.7 macrophage cells. Panel A shows the immunofluorenscence images for protein expression of IL-1β and Panel B shows the corresponding mRNA data. The relative mRNA level was quantified by scanning densitometry and normalized to β-actin mRNA. Note the up-regulated protein and mRNA expression of IL-1β by LPS is suppressed by different concentrations of resveratrol; however, in cells pretreated with PI3-K inhibitor wortmannin, the suppressive effect of resveratrol is abrogated. The values shown are mean ± SEM of data from three independent experiments. ^#^Significant compared with control alone, *p*<0.05. ^*^Significant compared with LPS alone, *p*<0.05. ^Δ^Significant compared with resveratrol + LPS, *p*<0.05.

To investigate whether resveratrol represses the production of TNF-α and IL-1β and whether PI3-K is involved in this process in RAW 264.7 cells, cells were stimulated with LPS (5 µg/ml; 6 h) in the presence or absence of resveratrol (1, 5, and 10 µM). After treatment with LPS, the protein levels of the cytokines in RAW 264.7 cells were evaluated by radioimmunoassay and immunofluorescence labeling which showed that TNF-α as well as IL-1β in the supernantant and immunoexpression was noticeably enhanced by LPS stimulation. Pre-treatment with resveratrol resulted in a drastic decrease the levels of TNF-α and IL-1β released into the medium and the immunoexpression levels of TNF-α and IL-1β. However, in RAW 264.7 cells subjected to pretreatment with 1 µM wortmannin for 1 h, TNF-α and IL-1β release and immunofluorescence intensity were obviously increased (Figures 4C and 4D; Figures 7A and 8A).

To further investigate whether the inhibitory effect of resveratrol on TNF-α and IL-1β production is due to the reduced expression of cognate genes, the effect of resveratrol on mRNA expression of TNF-α and IL-1β were assessed in LPS-stimulated RAW 264.7 cells. As shown in Figures 7B and 8B, the mRNA expression of these inflammatory mediators was very low or hardly detectable in unstimulated RAW 264.7 cells. However, RAW 264.7 cells expressed high levels of TNF-α and IL-1β mRNA when stimulated with LPS (5 µg/ml; 6 h). More importantly, resveratrol (1, 5, and 10 µM) suppressed LPS-induced expression of these genes. In parallel to the double immunofluorescence labeling, pretreatment of RAW 264.7 cells with wortmannin significantly reversed resveratrol-downregulated levels of TNF-α and IL-1β mRNA (Figures 7B and 8B).

These results suggest that the inhibitory effect of resveratrol on the proinflammatory cytokines TNF-α and IL-1β protein and mRNA levels is due to activation of the PI3-K signaling pathway.

### PI3-K Inhibition Blocks the Ability of Resveratrol to Inhibit LPS-stimulated Phosphorylation of CREB, and Protects Against LPS-induced Activation in RAW 264.7 Cells

**Figure 9:**
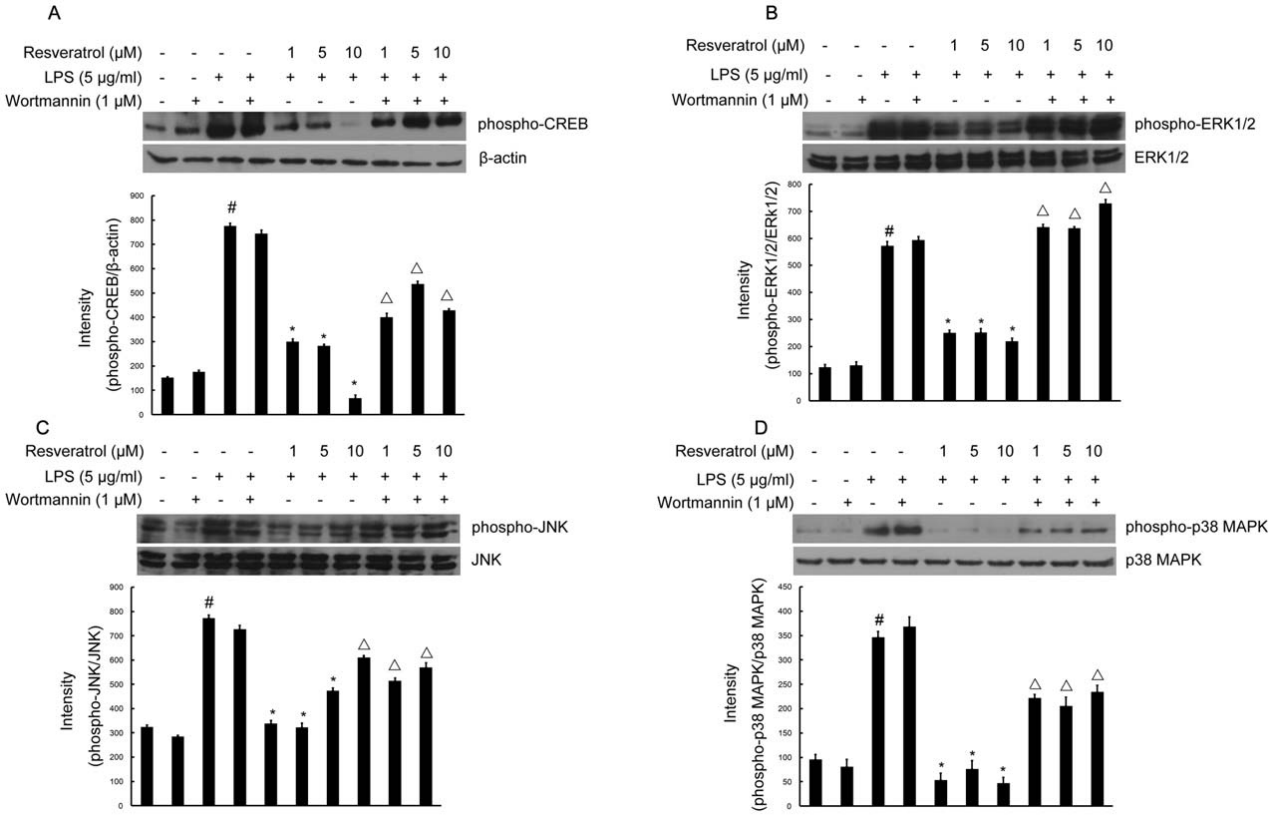
Inhibition of the phosphorylation of CREB, and MAPKs signaling by resveratrol is PI3-K-dependent during RAW 264.7 macrophage cells activation by LPS. Approximately 1×10^6^ cells/ml were seeded in six-well plates and incubated until 80% confluency. Cells were pre-treated with resveratrol (1, 5, and 10 µM) for 1 h in the absence or presence of wortmannin (1 µM), then exposed to LPS (5 µg/ml) for 30 min. Cell lysates (50 µg protein) were prepared and subjected to Western blot analysis by using antibodies specific for phosphorylated forms of CREB, ERK1/2, JNK and p38 MAPK (shown as phospho-IκB-α, etc.) as described in the methods. Equivalent loading of cell lysates was determined by reprobing the blots with anti-β-actin, total ERK1/2, JNK or p38 MAPK antibodies. The relative protein levels were quantified by scanning densitometry and normalized to β-actin, total ERK1/2, JNK or p38 MAPK. The values shown are mean ± SEM of data from three independent experiments. ^#^Significant compared with control alone, *p*<0.05. ^*^Significant compared with LPS alone, *p*<0.05. ^Δ^Significant compared with resveratrol + LPS, *p*<0.05.

To elucidate the mechanisms of resveratrol on the inhibition of expression of iNOS, COX-2, and proinflammatory cytokines in RAW 264.7 cells, we next determined whether resveratrol regulates the phosphorylation of CREB and whether such an effect, if any, is mediated through the PI3-K signaling pathway in RAW 264.7 cells. Treatmeng of cells with LPS (5 µg/ml) resulted in phosphorylation of CREB, whereas resveratrol (1, 5, and 10 µM) pretreatment inhibited LPS-stimulated phosphorylation of CREB. Addition of wortmannin (1 µM) suppressed the inhibitory action of resveratrol (Figure 9A).

These results indicate that wortmannin interferes with the inhibitory effects of resveratrol on LPS-activated phosphorylation. These data confirm that activation of PI3-K is essential for resveratrol-mediated inhibition of the phosphorylation of CREB.

### PI3-K Inhibition Reverses the Ability of Resveratrol to Block Phosphorylation of MAPKs Signaling in RAW 264.7 Cells

To further understand the molecular basis of resveratrol on the protection against LPS-induced activation in RAW 264.7 cells, we next examined whether the inhibition of phosphorylation of MAPKs by resveratrol, which are upstream signaling molecules in inflammatory reactions, are mediated via PI3-K signaling in LPS-stimulated RAW 264.7 cells. Western blot analysis was carried out using the phospho- or total forms of antibodies against the three MAPKs, ERK1/2, JNK, and p38 MAPK. Resveratrol at different concentrations 1, 5, and 10 µM significantly decreased the LPS-stimulated phosphorylation of ERK1/2 at 30 min, respectively, whereas it had no effect on the expression level of ERK1/2 in LPS-stimulated RAW 264.7 cells (Figure 9B). Likewise, resveratrol at all concentrations significantly suppressed the phosphorylation of JNK and p38 MAPK, respectively, but did not affect the expression levels of JNK and p38 MAPK in LPS-stimulated RAW 264.7 cells (Figures 9C and D). However, RAW 264.7 cells were pretreated with 1 µM wortmannin (1 h before) and, very interestingly, this reversed the effect of resveratrol-inhibited phosphorylation of ERK1/2, JNK and p38 MAPK (Figures 9B, C and D).

These results suggest that the ability of resveratrol to inhibit phosphorylation of MAPKs family is mediated by the PI3-K signaling pathway.

### PI3-K is Involved in Resveratrol-increased Phosphorylation of AMPK and Expression of SIRT1 in LPS Stimulated RAW 264.7 Cells

**Figure 10:**
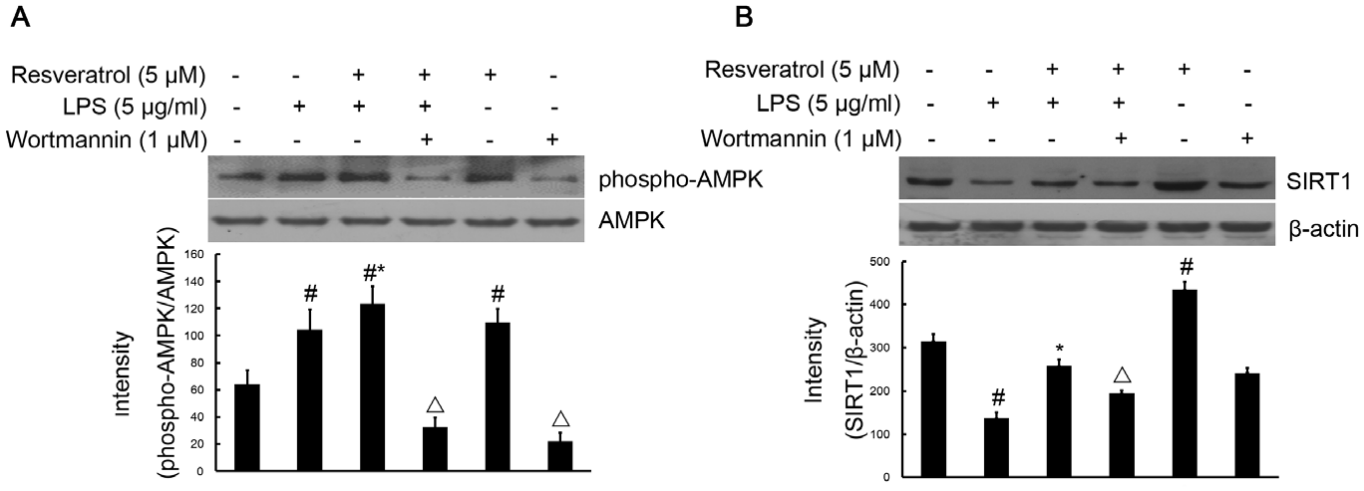
PI3-K is required for resveratrol-inhibited the phosphorylation of AMPK and the expression of the SIRT1 protein induced by LPS in RAW 264.7 macrophage cells. RAW 264.7 cells were pre-treated with resveratrol (5 µM) for 1 h in the absence or presence of wortmannin (1 µM), then exposed to LPS (5 µg/ml) for 40 min (Pane A) and 6 h (Panel B). Cell lysates (50 µg protein) were prepared and subjected to Western blot analysis by using antibodies specific for total AMPK, phosphorylated forms of AMPK (shown as phospho-AMPK), Sirt1 and β-actin as described in the methods. The values shown are mean ± SEM of data from three independent experiments. ^#^Significant compared with control alone, p<0.05. *Significant compared with LPS alone, p<0.05. ^Δ^Significant compared with resveratrol + LPS, p<0.05.

The effect of resveratrol on AMPK and SIRT1, which are upstream signaling molecules in inflammatory reactions, was examined in the LPS-stimulated RAW264.7 cells. We next determined whether resveratrol regulates phosphorylation of AMPK and expression of SIRT1, and whether the resveratrol effect is mediated through the PI3-K signaling pathway. Treatment of cells with LPS (5 µg/ml) slightly induced phosphorylation of AMPK (40 min) and inhibited expression of SIRT1 (6 h) in RAW264.7 cells, whereas resveratrol (5 µM) pretreatment further increased LPS-stimulated phosphorylation of AMPK and reversed LPS-inhibited expression of SIRT1. Addition of wortmannin (1 µM) suppressed the action of resveratrol (figures 10A and B).

These data confirm that activation of PI3-K/Akt is essential for resveratrol-mediated phosphorylation of AMPK and expression of SIRT1.

## Discussion

The pathophysiology of various inflammatory diseases is a complex process mediated by inflammatory and immune cells such as macrophages and monocytes. Because of the complexity of the pathophysiology of chronic inflammatory diseases, major efforts have focused on identifying novel anti-inflammatory drugs that prevent the proinflammatory process at the early stage of gene expression of key proinflammatory mediators and proinflammatory cytokines [31]. There is ample evidence, derived from some studies, showing that resveratrol down-regulates LPS-induced release of proinflammatory factors (NO, PGE2, iNOS and COX-2) and proinflammatory mediators (TNF-α and IL-1β) in RAW 264.7 macrophage cells pretreated with resveratrol [34], [35], [36], [37], [38]. In the present study showed that resveratrol (1, 5, and 10 µM) significantly inhibited the production of NO, PGE2, iNOS, COX-2, TNF-α and IL-1β in LPS-stimulated RAW 264.7 cells. Therefore, the data are consistent with previous findings that resveratrol inhibit proinflammatory mediators and proinflammatory factors, suggesting possible beneficial effects of resveratrol by attenuation of activation of RAW 264.7 macrophage cells and subsequent inflammatory neurotoxins. However, the signaling cascades that mediate the effects of resveratrol on proinflammatory factors production have not been previously characterized.

PI3-K is a class of phosphate kinase that has been implicated in cell signaling pathways that affect cellular death and longevity as well as many other processes that have medically important implications related to disease states [39]. The preferred substrate of class I PI3-K is phosphoinositide (4,5) bisphosphate (PIP2). Phosphorylation of PIP2 by PI3-K generates PIP3, which is an important second messenger that can promote cell survival, growth, protein synthesis, mitosis, and motility [40]. Recently, several lines of evidence suggest an involvement of PI3-K-linked cascades in the negative regulation of LPS-induced inflammatory responses *in vitro* and *in vivo* [31], [41], [42].The present study was undertaken to investigate whether these effects of resveratrol were mediated via PI3-K/Akt signaling pathways. We found that resveratrol activated the PI3-K pathway and induced Akt phosphorylation in a time-dependent manner in RAW 264.7 cells. Similar to resveratrol, LPS also induced Akt phosphorylation in RAW 264.7 cells. However, the levels of phospho-Akt increased 20, 40 and 60 min after the addition of LPS, specially increased significantly in 40 min. These findings are consistent with the studies [43], which showed that the LPS-induced activation of the PI3K/Akt pathway was slightly delayed relative to the activation of the MAPK pathways. Thus, it appears that the delay in PI3K/Akt activation initially allows LPS to activate MAPK and induce an acute inflammatory response, which is subsequently blunted and then shut down by LPS-induced Akt phosphorylation [31]. In contrast, when the cells were treated with resveratrol before LPS stimulation, Akt phosphorylation was elevated initially and further increased during LPS exposure. Hence, the proinflammatory response to LPS was suppressed by resveratrol from the beginning, resulting in inhibition of LPS-induced MAPKs activity and CREB gene expression. These anti-inflammatory effects of resveratrol in RAW 264.7 cells were abolished by the PI3K inhibitor wortmannin, indicating that they required PI3K/Akt activation.

We further used the PI3-K inhibitor wortmannin to demonstrate that resveratrol-mediated activation of PI3-K is necessary for resveratrol-induced suppression of proinflammatory mediators and proinflammatory cytokines production in LPS-stimulated RAW 264.7 cells. The present demonstration that PI3-K inhibition prevents the effects of resveratrol on NO, PGE2, iNOS, COX-2, TNF-α and IL-1β production is reminiscent of the recent report demonstrating that icariin-induced inhibition of proinflammatory cytokines expression in LPS-stimulated RAW 264.7 cells is dependent on PI3-K/Akt activation [32]. Additionally, inhibition of PI3-K induced the expression of proinflammatory mediators and proinflammatory cytokines production in LPS-stimulated microglial cells, cardiomyocytes and human lung epithelial adenocarcinoma cells [44], [45], [46]. These reports further strengthen our findings and demonstrate an important role for PI3-K/Akt signaling in the regulation of proinflammatory cytokines production in various cell types. The transcriptional regulation of iNOS, COX-2 and inflammatory cytokines, such a s TNF-α and IL-1β, is a tightly controlled event. A variety of transcription factors, including CREB, are known to be involved in the transcriptional regulation of these inflammatory mediators [47]. The present results have shown that phosphorylation level of CREB, markedly increased by LPS stimulation in RAW 264.7 cells, was effectively reversed by resveratrol treatment in a PI3-K-dependent manner.

MAPKs are mainly composed of three well-characterized subfamilies including ERK1/2, JNK and p38 MAPK, which elicit many cellular functions in physiological conditions and pathological diseases [48]. MAPKs family has been shown to play important roles in LPS-induced expression of iNOS, COX-2, and proinflammatory cytokines in many types of cells [12], [49], [50], [51]. In resveratrol-attenuated levels of proinflammatory mediators and proinflammatory cytokines production, a pivotal role for three MAPK members in signaling transduction of the various cells was identified [26], [52], [53], [54]. To further investigate the signaling pathway by which resveratrol inhibits LPS-induced phosphorylation of MAPKs family and whether PI3-K/Akt is involved in this process in RAW 264.7 cells, we examined the effect of resveratrol on activation (phosphorylation) of three MAPKs induced by LPS in RAW 264.7 cells. As expected, LPS increased activation of MAPKs, including ERK1/2, JNK, and p38 MAPK, within 30 min after stimulation. Remarkably, resveratrol decreased the LPS-induced activation of MAPKs along with reduced expression NO, PGE2, iNOS, COX-2, and proinflammatory cytokines. There are reports in the literature have showed that resveratrol had no effect on LPS-stimulated phosphorylation of ERK1/2 and p38 in microglia, astrocytes and non-myeloid AR42J pancreatic cells, but slightly inhibited LPS-stimulated phosphorylation of JNK in astrocytes [55], [56]. The reports have suggested that the results occurs at higher concentrations of resveratrol and differences in cell origin; additionally, PI3K inhibition significantly reversed the effect of resveratrol-inhibited phosphorylation of ERK1/2, JNK and p38 MAPK. Thus, our data provide evidence that the PI3K/Akt pathway plays a critical role in attenuating LPS-induced acute inflammatory responses in RAW 264.7 cells that this pathway serves as a negative regulator of LPS-induced MAPKs activation and expression of proinflammatory mediators and proinflammatory cytokines.

There is accumulating evidence that both AMPK and SIRT1 have been suggested to mediate the inhibition of resveratrol on LPS-stimulated proinflammatory cytokines release [57.58.59]. However, the precise mechanisms by which resveratrol activate AMPK and SIRT1 were not established, but it is possible that resveratrol may all trigger a common molecular signaling cascade that ultimately activates AMPK and SIRT1. The present results have shown that the phosphorylation of AMPK and the expression of SIRT1 were effectively increased by resveratrol treatment in a PI3-K/Akt -dependent manner in LPS-stimulated RAW264.7 cells. Meanwhile, recent a study indicated that SIRT1 as a positive modulator of insulin signaling in muscle cells through PI3-K, and this mechanism appears to be conserved from C. elegans through humans [60]. Together, these data suggest that resveratrol may exert dependent effects on AMPK/SIRT1 and PI3-K/Akt pathway, with subsequent cross-talk between the two signaling pathway. Future studies are needed to elucidate a detailed mechanism of action of resveratrol on AMPK/SIRT1 signaling.

**Figure 11:**
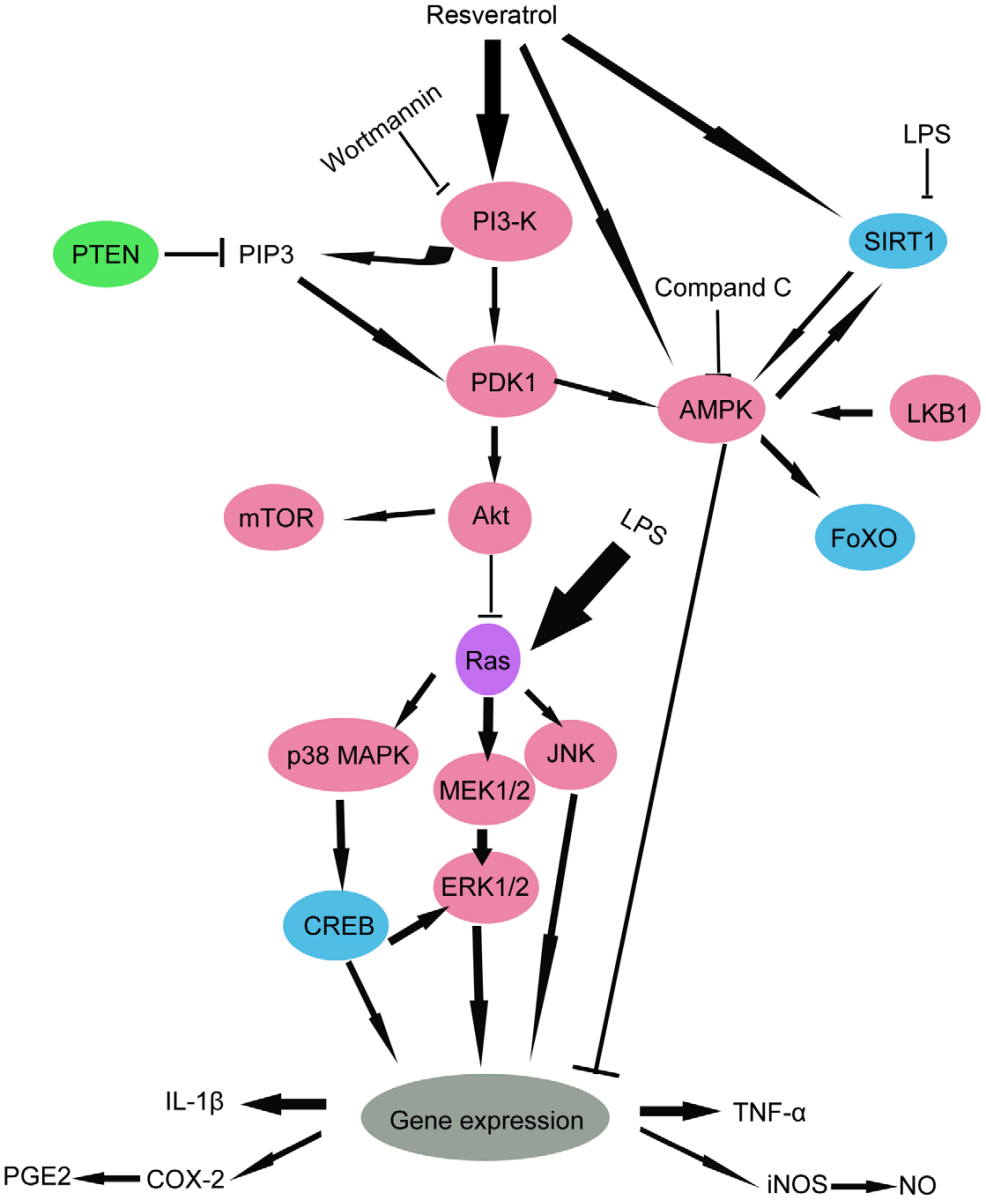
A schematic diagram showing the interlinks of PI3-K signaling pathway.

This report provides the first evidence demonstrating that PI3-K/Akt activation is an important signaling in resveratrol-mediated activation of AMPK phosphorylation and SIRT1 expression, and inhibition of MAPKs activation, proinflammatory mediators and proinflammatory cytokines production in response to LPS in RAW 264.7 cells as depicted semi-diagrammatically in figure 11. Thus, the PI3-K/Akt pathway is one of the signaling pathways by which resveratrol exerts its cytoprotective effects. These findings further our knowledge regarding the pathways used by resveratrol in exerting its protective effects and support our belief that resveratrol may hold future clinical promise in the development of novel therapeutic strategies for the treatment of the inflammatory diseases. On the hand, the *in vivo* biological effects of resveratrol appear strongly limited by its low bioavailability, which is a barrier to the development of therapeutic applications, in particular after oral administration [61], [62]. Therefore, the future research should aim to the development of innovative formulation strategies that are able to overcome each of the physicochemical, pharmacokinetic and metabolic limitations of resveratrol.

## Methods

### Cells and Treatments

The immortalized mouse macrophage cell line RAW 264.7 obtained from American Type Culture Collection (Livingstone, MT, USA). Cells were cultured in Dulbecco’s modified Eagle’s medium (DMEM; Gibco/BRL, Gaithersburg, MD, USA) containing 2% fetal bovine serum (Hyclone, Logan, UT, USA) and antibiotics (100 IU/ml penicillin and 100 µg/ml streptomycin; Sigma, St. Louis, MO, USA) at a density not exceeding 5×10^5^ cells/ml and maintained at 37°C in a humidified incubator with 5% CO_2_. To harvest RAW 264.7 cells, cells were trypsinized (0.25% trypsin/EDTA in phosphate-buffered saline (PBS); Sigma, St. Louis, MO, USA), then centrifuged (400 g for 10 min) and resuspended in serum-free DMEM. Cells were counted with a hemocytometer and trypan blue staining (0.4% trypan blue in PBS; Sigma) showed more than 98% of the cells retained viability. Cells (approximately 1×10^6^ cells/ml) were seeded in six-well plates before being subjected to treatments. Resveratrol (Purity >99%; Sigma) at 1, 5, and 10 µM was added 1 h before LPS (5 µg/ml) (from *Escherichia coli,* Sigma) stimulation. This time point was chosen to minimize the possibility of any direct interactions between resveratrol and LPS. Wortmannin (1 µM; Sigma), a specific PI3-K inhibitor, was used to determine if PI3-K/Akt signaling pathway might be involved in resveratrol’s action on RAW 264.7 cells. Cell incubations ranged from 20 min–24 h, as indicated in the text.

### Cell Cytotoxicity Test

RAW 264.7 cells were seeded in a 24-well dish (1×10^6^ cells/ml) before being exposed to resveratrol (1, 5, and 10 µM), resveratrol with LPS (5 µg/ml), resveratrol with wortmannin (1 µM), or resveratrol with LPS + wortmannin for 24 h. MTT solution (0.5 mg/ml) was then added to each well and the cells were incubated for 2 h at 37°C and in 5% CO_2_. Subsequently, the supernatant was removed and the formation of farmazan was solubilized with dimethyl sulfoxide (DMSO) and measured at 540 nm with a microplate reader.

### Assay of NO Production

RAW 264.7 cells (1×10^6^ cells/ml per well in a 24 well plate) were pretreated with resveratrol (1, 5, and 10 µM) for 1 h and stimulated with LPS (5 µg/ml) in the presence of wortmannin (1 µM) for 12 h. NO production was monitored by measuring the nitrite content in culture medium as previously described [31]. Culture supernatants were mixed with an equal volume of Griess reagent (0.1% N-1-naphthylethylenediamine dihydrochloride and 1% sulphanilamide in 5% phosphoric acid) and incubated at room temperature for 10 min. Absorbance was measured at 550 nm in a microplate reader. Sodium nitrite was used as a standard.

### Determination of PGE2 Production

Various treated RAW 264.7 cells were plated in 24-well plates at a density of 1×10^6^ cells/ml for 12 h. The PGE2 concentration in the culture supernatants was quantified using a competitive enzyme immunoassay kit (R&D Systems Inc., MN, USA) in accordance with the manufacturer’s instructions. The production of PGE2 was measured relative to that observed after control treatment.

### Measurement of TNF-α and IL-1β by Radioimmunoassay

Various treated RAW 264.7 cells were plated in 24-well plates at a density of 1×10^6^ cells/ml for 12 h. The contents of the proinflammatory cytokines TNF-α and IL-1β in the cell-free supernatant of RAW264.7 cells culture medium were measured with specific radioimmunoassay kits (Beijing East Asia Institute of Immunology, Beijing, China). The concentrations of TNF-α and IL-1β were determined by interpolation from a standard curve. The cytokine levels were normalised based on the protein concentration of individual dishes, and protein concentration was determined by the Lowry method.

### Double Immunofluorescence Labeling

RAW 264.7 cells derived from various treatments were fixed with 4% paraformaldehyde in 0.1 M phosphate buffer (PB) for 15 min. After rinsing with PBS, the coverslips with adherent cells were used for double immunofluorescence labeling. RAW 264.7 cells were incubated with DAPI (dilution 1∶50,000; Sigma) plus goat anti-mouse iNOS (dilution 1∶500; Santa Cruz Biotechnology, Santa Cruz, CA, USA), goat anti-mouse COX-2 (dilution 1∶500; Santa Cruz Biotechnology), goat anti-rabbit TNF-α polyclonal antibody (dilution 1∶500; Chemicon, Temecula, CA,USA), goat anti-rabbit IL-1β (dilution 1∶500; Chemicon). Subsequently, the cells were incubated with TRITC-conjugated secondary antibody (Santa Cruz Biotechnology) for 1 h at room temperature. For negative controls, a set of culture slides was incubated under similar conditions without the primary antibodies. All images were captured with a fluorescence microscope (80i; Nikon, Tokyo, Japan). The results are representative of three independent experiments.

### Reverse Transcription-polymerase Chain Reaction (RT-PCR) Analysis

Total RNA was prepared from RAW 264.7 cells by using the Trizol® reagent (Invitrogen Corporation, Carlsbad, CA, USA) according to the manufacturer’s protocol. Total RNA was reverse-transcribed by using the Superscript™-III kit (Invitrogen) with 2.5 µg total RNA and oligo dT. Primer sequences were as follows: iNOS, sense: 5′- CTGCAGCACTTGGATCAGGAACCTG -3′, antisense: 5′- GGGAGTAGCCTGTGTGCACCTGGAA -3′; COX-2, sense: 5′-TTGAAGACCAGGAGTACAG C-3′, antisense: 5′-GGTACAGTTCCATGACATCG-3′; TNF-α, sense: 5′-CGTCAGCCGATTTGC TATCT-3′, antisense: 5′-CGGACTCCGCAAAGTCTAAG-3′; IL-1β, sense: 5′-GCCCATCCTCTG TGACTCAT-3′, antisense: 5′-AGGCCACAGGTATTTTGTCG-3′; β-actin, sense: 5′-AGCCATGTACGTAGCCATCC-3′, antisense: 5′-GCTGTGGTGGTGAAGCTGTA-3′. PCR amplification of the resulting cDNA template was conducted by using the following conditions for 45 (TNF-α IL-1β and β-actin), 36 (COX-2) or 27 (iNOS) cycles. After an initial denaturation step at 95°C for 15 min, temperature cycling was initiated. Each cycle consisted of denaturation at 94°C for 15 sec, annealing at 60°C for 25 sec, and elongation at 72°C for 20 sec (TNF-α IL-1β and β-actin). For COX-2, after an initial denaturation step at 95°C for 5 min, temperature cycling was initiated. Each cycle consisted of denaturation at 94°C for 30 sec, annealing at 57°C for 45 sec, and elongation at 72°C for 30 sec. For iNOS, after an initial denaturation step at 95°C for 5 min, temperature cycling was initiated. Each cycle consisted of denaturation at 94°C for 45 sec, annealing at 60°C for 45 sec, and elongation at 70°C for 1 min. PCR products were analyzed on 1% agarose gels and stained with 1 mg/ml ethidium bromide. Images were captured with a Gel Doc 2000 image analyzer (Bio-Rad, Richmond, CA, USA). The results are representative of three independent experiments.

### Western Blot Analysis

RAW 264.7 cells were plated overnight in 6 well plates at a density of 1×10^6^ cells per plate. The cells were further incubated in a medium without 10% FBS for at least 1 h before treatments. Stimulated cells were harvested with ice-cold PBS and centrifuged at 16 000×*g* for 5 min at 4°C. Cells were lysed in ice-cold lysis buffer [62.5 mM Tris–HCl, pH 6.8, 25% glycerol, 2% sodium dodecyl sulphate (SDS), 0.01% bromphenol blue and 5% β-mercaptoethanol]. Cell lysates were centrifuged at 16 000×*g* for 5 min at 4°C, then the supernatants were collected. Protein content was determined by using the BCA protein assay (Pierce, Rockford, IL, USA). Equal amounts of protein (50 µg) were loaded per lane onto 10% SDS-polyacrylamide gel electrophoresis (SDS-PAGE) and transferred onto immunoblot polyvinylidene difluoride membranes (Chemicon). The membranes were blocked with 5% non-fat milk in Tris-buffered saline containing 0.1% Tween 20 (TBS-T) for 2 h at room temperature and incubated separately with goat anti-rabbit antibodies for Akt and phospho-Akt, ERK1/2 and phospho-ERK1/2, JNK and phospho-JNK, p38 MAPK and phospho-p38 MAPK, AMPK and phospho-AMPK, SIRT1, phospho-CREB and β-actin antibodies (1∶1000 dilution; Cell Signaling Technology, Danvers, MA, USA) that recognize different molecules under study for overnight at 4°C. The membranes were then washed three times for 15 min with TBS-T, and incubated with a 1∶2000 dilution of horseradish peroxidase-coupled secondary antibodies (Santa Cruz Biotechnology) for 2 h at room temperature. Blots were again washed three times for 5 min each in TBS-T and developed by the ECL® detection system (Santa Cruz Biotechnology). Membranes were exposed to Fuji Medical X-Ray Film (Fuji Photo Film Co., Ltd, Karagawa, Japan).

### Statistical Analysis

Statistical analysis of the data was carried out by one way analysis of variance (ANOVA) followed by Scheffe’s post hoc test, using SPSS (SPSS Inc., Chicago, IL, USA). Summary data are shown as mean ± SEM (standard error of mean) obtained from three independent experiments. Values of *p*<0.05 were considered significant. (^#^Significant compared with control alone, *p*<0.05; ^*^Significant compared with LPS alone, *p*<0.05; ^Δ^Significant compared with resveratrol + LPS, *p*<0.05).
